# Fracture resistance of teeth obturated with Gutta percha and Resilon: An *in vitro* study

**DOI:** 10.4103/0972-0707.66712

**Published:** 2010

**Authors:** Suheel Manzoor Baba, Shiban I Grover, Varsha Tyagi

**Affiliations:** Department of Conservative Dentistry and Endodontics, Subharti Dental College, Meerut-250 002, India

**Keywords:** Apexification, artificial barrier, calcium sulfate, mineral trioxide aggregate

## Abstract

**Aim:**

The aim of this study was to evaluate and compare the fracture resistance of endodontically treated teeth filled with gutta percha and Resilon.

**Materials and Methods:**

A total of 60 freshly extracted single rooted teeth are selected and their anatomical crown removed at the CEJ. All samples were instrumented with the Step-back technique. Samples were randomly divided into three groups of 20 samples each: Group A obturated by lateral condensation with gutta percha and AH plus sealer, Group B obturated by lateral condensation with Resilon; Group C recieved no filling. Restored teeth were subjected to compressive loading in a universal testing machine. The results of fracture resistance recorded and statistical analysis done.

**Results:**

The mean and SD values for the groups are as follows: Group A-536.555 ± 128.816, Group B- 885.943 ± 194.410 and Group C- 591.066 ± 68.97. It was seen that samples of Group B showed the highest fracture resistance followed by Group C. Group A showed the least fracture resistance.

**Conclusion:**

The results of this study showed that filling the canals with Resilon increased the *in vitro* resistance to fracture of endodontically treated single canal teeth when compared with gutta percha.

## INTRODUCTION

The primary objective of root canal therapy is complete obturation of the root canal system. Teeth with pulpal involvement are endodontically treated by cleaning and shaping the root canal system, followed by total obturation of that space. Once a fluid-tight seal is established, any inflammatory reaction initiated or promoted by noxious material from within the canal should cease. This allows healing of periradicular pathosis. However, if the canal is not completely cleaned and sealed, inflammation and infection may continue.[1]

Obturation is one of the most critical step in root canal therapy. Hence, it should be performed according to the highest clinical standards. Gutta percha used in conjunction with root canal sealers may have been the best combination available to date and is seen as the gold standard of root canal fillings. Despite its many advantages and having achieved the status of a time-honored material, gutta percha still has its limitations like its inability to strengthen root as it does not bond to dentin. Although few materials have seriously challenged gutta percha in the majority of filling situations, research continues to find alternatives that may seal better and mechanically reinforce compromised roots.[2,3]

In 2004, a new obturation system Resilon was introduced containing Resilon along with resin-based sealer. Resilon performs in a similar way to gutta percha, has the same handling properties and can be heat softened or dissolved with solvents such as chloroform during retreatment procedures. A real seal/epiphany sealer is a dual-cure resin composite sealer which is used in conjunction with Resilon points.[4] The Resilon System is expected to form a monoblock within the canal space, whereby the core (Resilon) is bonded to the sealer (Epiphany) and the resulting complex is bonded to the root dentine by the resin-based primer. Such a monoblock has been suggested to reduce bacterial ingress pathways and strengthen the root to some extent[5]. This study was done to evaluate the fracture resistance of teeth obturated with gutta percha and with Resilon.

## MATERIALS AND METHODS

Sixty freshly extracted single rooted human mandibular premolar teeth freshly extracted for orthodontic purpose, with a root length of at least 14 mm were selected for the study and stored in saline.

The samples were cleaned and disinfected with sodium hypochlorite solution and the crowns sectioned at the cementoenamel junction. A#15K file was advanced in the canal until the tip of the instrument was visible at the apex and the stop was placed at the coronal reference point i.e. the cementoenamel junction. The working length was determined by subtracting 1 mm from the previous length.

All the teeth were instrumented with K-files while 5 ml of 5.25% sodium hypochlorite was used to irrigate the preparation after each instrumentation. The apical preparation was done until #40 file and the body of the canal was prepared by the step back technique until #55 K-file. All samples received a final flush with 17% EDTA. The prepared canals were then dried with sterile paper points. The samples were randomly divided into three groups of 20 teeth as follows:

### Group-A

Canals obturated with gutta percha and AH-plus sealer. Dentinal walls of the root canal were coated with AH plus sealer with the help of a lentulo spiral. Lateral condensation was accomplished using a size 40 gutta-percha master cone, dipped in AH plus sealer according to the manufacturer’s instructions. After placement of the master cone at the appropriate working length, accessory cones of gutta percha dipped in AH plus sealer were used for the lateral compaction with the help of spreader.

### Group-B

Canals obturated with Resilon points and Resilon sealer. Root canal walls were coated with Resilon sealer with the help of a lentulo spiral. Lateral condensation was accomplished using a size 40 Resilon master cone dipped in resin sealer according to the manufacturer’s instruction. After placing the master cone to the working length, Resilon accessory cones dipped in resin sealer were laterally condensed with the help of spreader. Excess material was seared off and condensed vertically with a plugger 1 mm below the canal opening. After this procedure, the material in the root canal was cured with visible light for 30 s. Canal openings were sealed with Cavit.

### Group-C

Canals received no obturation. This group received no obturation, and the root canal opening of all the samples were sealed with Cavit.

### Storage of samples

All samples were stored in 100% humidity at 37°C for 1 week in an incubator to simulate *in vivo* conditions ensuring correct setting of sealing material.

### Preparation for mechanical testing

The apical root ends were embedded individually in copper moulds (height = 20 mm, length = 20mm, width = 20 mm) with acrylic resin, leaving 8mm of each root exposed. The temporary material was removed and the root canal access was shaped with the help of carbide bur to accept the loading fixture. The blocks were mounted with the vertically aligned roots in the testing machine one at a time.

A loading fixture with a spherical tip(r = 2 mm) was mounted and aligned with the center of the canal opening of each specimen. Each specimen was subjected to load at a crosshead speed of 1.0 mm/min until the root fractured. This is the point at which a sharp and instantaneous drop greater than 25% of the applied load was observed. The test was terminated at this point and the force required to fracture the roots was recorded in Newton’s.

## RESULTS

The table shows mean and standard deviation for each experimental group. ANOVA revealed a significant difference between the groups. The Resilon group displayed higher mean fracture and the gutta percha group displayed lower mean fractures load values than did the unfilled control group [Table 1].

**Table 1:** Standard and mean deviation

Groups	Mean ± S.D	S.E.M.	Min. (In Newtons)	Max. (In Newtons)
A	536.555 ± 128.816	28.804	290.83	822.39
B	885.943± 194.410	43.471	633.39	1520.45
C	591.066 ± 68.97	15.424	454.63	856.36

On comparing mean and SD values as shown in Table 2 (Group A-536.555 ± 128.816, Group B- 885.943 ± 194.410 and Group C- 591.066 ± 68.97) it was seen that samples of Group B showed the highest fracture resistance followed by Group C. Group A showed the least fracture resistance.

**Table 2:** One-way ANOVA (analysis of variance)

Source of variance	D.F.(Degree of freedom)	S.S.(Sum of squares)	M.S.S.(Mean of squares)	F-ratio (Calculated)	F_tab_	*P* value
Between groups	2	1391521.9	695760.95	35.0295	3.14 (5)	< 0.05
Residual	57	1132142.11	19862.142		4.97 (1)	< 0.01
Total	59	2523664				

Figures in parentheses are in percentage

## DISCUSSION

Gutta percha is gold standard in the root filling materials but limitations of gutter percha i.e. inability to reinforce endodontically treated roots and coronal microleakage has lead to the development of an alternative.[6,7]

Few studies have evaluated the potential of using dentin-bonding agents and resins as obturation materials in nonsurgical root canal treatment. Reasons for not using resins have centered on questionable results, difficult and unpredictable methods of delivery into the root system and the inability to re-treat the canal if necessary.[8,9]

Recently introduced Resilon is a thermoplastic synthetic polymer. Resilon is based on polymers of polyester and contains bioactive and highly radiopaque fillers. The polymer has an improved flexural strength and when used in conjunction with a resin-based sealer offers improved bonding potential, as compared to gutta percha. Primary components of Resilon are (1) Resilon core material containing bioactive glass, Bismuth oxychloride and Barium sulfate. (2) Resin sealer is a dual cure, resin-based composite sealer. The resin matrix is composed of bisphenol-A-glycidyldimethacrylate (BisGMA), ethoxylated BisGMA, urethanedimethacrylate (UDMA), and hydrophilic difunctional dimethacrylates. It contains fillers of calcium hydroxide, barium sulfate, barium glass, and silica. The total filler content is approximately 70% by weight. (3) Self-etch primer that contains sulfonic acid-terminated functional monomer, 2-hydroxyyethyl methacrylate (HEMA), water and a polymerization initiator. HEMA enhances the bonding of resin to dentin.[6,7]

The results of the present study demonstrated that root canal obturated with Resilon resulted in higher resistance to fracture when compared with the roots obturated with a gutta percha/AH plus sealer and also with the instrumented but unfilled roots. Resilon is a synthetic polymer; resin sealer attaches to it as well as to bonding agent or primer used to penetrate into dentinal tubules. As a result, a monoblock is formed (consisting of Resilon core material, Resin sealer, bonding agent/primer and dentin).[7,10] Another reason of Resilon being a better obturating material could be that the removal of smear layer by EDTA after biomechanical preparation may have allowed the root canal filling material and root canal sealers to contact the canal wall and penetrate in the dentinal tubules which may increase the strength of roots.[11]

Although gutta percha has been the standard obturating material used in root canal treatment, but it does not reinforce endodontically treated roots owing to its inability to achieve an impervious seal along the dentinal walls of the root canal as stated by Aptekar *et al*.[10] Gutta percha does not show increased resistance to internally generated stresses in root canal because it does not chemically bond to the dentin wall i.e. does not form the monoblock system. According to Teixeira *et al*,[7] gutta percha does not from a monoblock even with the use of a resin-based sealer such as AH plus, because the sealer does not bind to gutta percha. Moreover, the sealer tends to pull away from the gutta percha on setting. Also the wedging forces applied by the spreader during later condensation decreased the fracture resistance in the gutta percha group. This is in good agreement with the study conducted by Piskin *et al*.^12^ which state that the spreader size larger than 25 number causes significant reduction in fracture resistance of roots and another study conducted by Lertchirakarn *et al*, 13 which state that fracture can result from excessive lateral condensation forces during the root filling. Saw and Messer[10] reported that a higher value of apical strain than coronal strain was generated during lateral compaction, probably because of the reduced thickness of dentin in the apical portion of the root and the greater wedging effect of the spreader tip in the narrower part of the canal.

The control group in which the teeth were instrumented but not obturated has shown less resistance to fracture than the Resilon group, as there was no obturating material to reinforce the teeth, and the control group showed more resistance to fracture than teeth obturated with the gutta percha group, as there were no forces imparted in the teeth due to lateral condensation.

## CONCLUSION

Within the limitations of this study analysis of data yielded:

1. Highest resistance to fracture was shown by Group B (Resilon) followed by Group C (Control).
2. Group A (gutta Percha with AH plus sealer) showed least resistance to fracture.
3. On inter-group comparison statistically significant difference was seen between Resilon and gutta percha and also between the Resilon and control group.

Resilon demonstrated high fracture resistance values and could be an alternative to the conventional gutta percha.
